# Genome Sequence of Rotavirus A from a Florida Racing Pigeon (*Columba livia domestica*)

**DOI:** 10.1128/mra.01149-21

**Published:** 2022-03-14

**Authors:** Rowan A. Basham, Jennifer Dill-Okubo, Kuttichantran Subramaniam, Thomas B. Waltzek, Pedro H. O. Viadanna

**Affiliations:** a Department of Infectious Diseases and Immunology, College of Veterinary Medicine, University of Florida, Gainesville, Florida, USA; b Emerging Pathogens Institute, University of Florida, Gainesville, Florida, USA; c Bronson Animal Disease Diagnostic Laboratory, Florida Department of Agriculture and Consumer Services, Kissimmee, Florida, USA; KU Leuven

## Abstract

The complete coding sequence of a rotavirus A strain was determined from a dead racing pigeon in Florida. It was found to be most closely related to a rotavirus A strain isolated from a dead racing pigeon in California.

## ANNOUNCEMENT

Pigeons (*Columba livia domestica*) are susceptible to a variety of parasitic, bacterial, and viral diseases (1). The genus *Rotavirus* (family *Reoviridae*) includes nine species, namely, rotaviruses A to D and F to J (2, 3). Pigeons infected with rotavirus A (RVA) have been reported in the United States (California), Australia, Denmark, Belgium, Hungary, and Germany (4–9). The RVA genome is composed of 11 segments of double-stranded RNA, which encode six structural proteins (VP1 to VP4, VP6, and VP7) and six nonstructural proteins (NSP1 to NSP6) (10, 11). The genotype of each rotavirus segment is determined based on nucleotide cutoff values (12, 13). This genotype classification system follows the notation Gx-P[x]-Ix-Rx-Cx-Mx-Ax-Nx-Tx-Ex-Hx for the genes *VP7-VP4-VP6-VP1-VP2-VP3-NSP1-NSP2-NSP3-NSP4-NSP5/6*, respectively (12, 13).

A 2-year-old Florida racing pigeon was submitted to the state diagnostic laboratory for necropsy. At necropsy, the intestine was distended, flaccid, and congested. Frozen intestinal tissue was sent to the Wildlife and Aquatic Veterinary Disease Laboratory in Gainesville, Florida, for viral discovery. RNA from the intestinal tissue was extracted using an RNeasy minikit (Qiagen) and served as the template for the generation of a cDNA sequencing library using a NEBNext Ultra directional RNA library preparation kit (New England Biolabs). The library was sequenced using a MiSeq Reagent kit v3 (600-cycle) on a MiSeq sequencer (Illumina). A total of 2,955,154 paired-end reads with an average read length of 240 bp were assembled *de novo* in SPAdes v3.13.0 (14) with default parameters. BLASTN searches of the resulting contigs against the National Center for Biotechnology Information (NCBI) nonredundant nucleotide database identified the nearly complete coding sequences for all 11 segments of a unique RVA strain. The 5′ ends of the coding sequences of segments 2 and 4 were determined using a 5′ rapid amplification of cDNA ends (RACE) PCR kit (Roche Diagnostics) and Sanger sequencing of the resulting amplicons. Gaps within segments 2 and 3 were amplified by reverse transcription PCR followed by Sanger sequencing. The total length of the complete coding sequences of the 11 segments was 17,794 bp, with a GC content of 34.98%. The average coverage was 22 reads/nucleotide.

**FIG 1:**
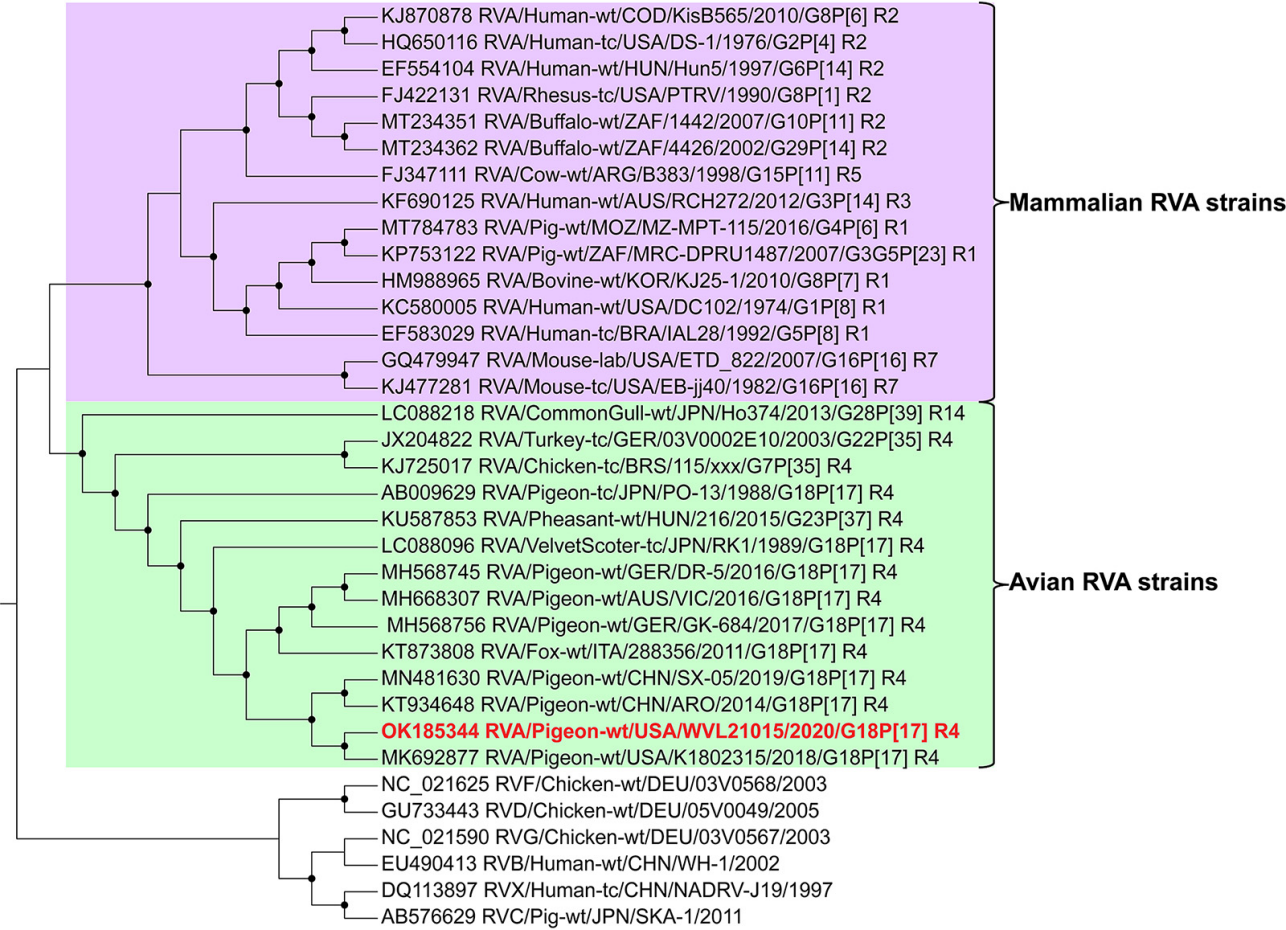
Maximum likelihood cladogram depicting the relationships of the RVA identified from a Florida racing pigeon (strain WVL21015, highlighted in red) to other rotaviruses, based on the nucleotide sequence alignment of the *VP1* gene. The rotaviruses are indicated by GenBank accession number, official rotavirus nomenclature, and genotype. The bracket highlighted in purple includes mammalian RVA strains, and the bracket highlighted in green includes avian rotavirus A strains. All nodes with black circles are supported by bootstrap values of >80%. The tree was rooted with non-RVA species.

BLASTN analysis of each of the 11 complete coding sequences showed the greatest identity (99.6 to 99.9%) to RVA/Pigeon-wt/USA/K1802315/2018/G18P[17], which was isolated from a dead racing pigeon in California, with genotype G18-P[17]-I4-R4-C4-M4-A4-T4-N4-E19-H4 (9) (Table 1). A maximum likelihood phylogenetic analysis, based on the nucleotide alignment of the *VP1* gene for 35 rotaviruses, was performed in IQ-TREE v1.4.4 (15) with 1,000 nonparametric standard bootstraps. The RVA strain (RVA/Pigeon-wt/USA/WVL21015/2020/G18P[17]) from the Florida racing pigeon grouped within the avian RVA genotype R4 (VP1) as the closest relative of the aforementioned RVA strain isolated from a California racing pigeon (Fig. 1). Using the Rotavirus Classification Tool (16), the complete genotype of the Florida racing pigeon RVA was determined to be identical to that of the California racing pigeon RVA (G18-P[17]-I4-R4-C4-M4-A4-T4-N4-E19-H4).

**TABLE 1:** BLASTN results for all 11 complete coding sequences of the rotavirus A strain from a Florida racing pigeon

Segment no.	Protein name	GC content (%)	Coding sequence length (bp)	GenBank accession no.	Data for top BLASTN match			
					BLASTN description	Genotype	Identity (%)	GenBank accession no.
1	VP1	32.7	3,273	OK185344	RVA isolate K1802315 segment 1	R4	99.6	MK692877
2	VP2	37.7	2,694	OK185345	RVA isolate K1802315 segment 2	C4	99.7	MK692878
3	VP3	32	2,490	OK185346	RVA isolate K1802315 segment 3	M4	99.8	MK692879
4	VP4	35.1	2,313	OK185347	RVA isolate K1802315 segment 4	P[17]	99.6	MK692880
5	NSP1	34.8	1,731	OK185348	RVA isolate K1802315 segment 5	A4	99.6	MK692881
6	VP6	38.4	1,194	OK185349	RVA isolate K1802315 segment 6	I4	99.8	MK692882
7	NSP3	36	921	OK185350	RVA isolate K1802315 segment 7	T4	99.9	MK692883
8	NSP2	34.4	948	OK185351	RVA isolate K1802315 segment 8	N4	99.6	MK692884
9	VP7	35.9	990	OK185352	RVA isolate K1802315 segment 9	G18	99.8	MK692885
10	NSP4	34.3	510	OK185353	RVA isolate K1802315 segment 10	E19	99.6	MK692886
11	NSP5	33.3	504	OK185354	RVA isolate K1802315 segment 11	H4	99.8	MK692887

Our study confirms that RVA was present in a population of Florida racing pigeons. Further study is needed to determine the distribution of pigeon RVA G18P[17] in Florida and its potential impact on the global industry.

### Data availability.

The genome and raw sequence data for RVA strain RVA/Pigeon-wt/USA/WVL21015/2020/G18-P[17]-I4-R4-C4-M4-A4-T4-N4-E19-H4 have been deposited in the NCBI GenBank and Sequence Read Archive (SRA) databases under accession numbers OK185344, OK185345, OK185346, OK185347, OK185348, OK185349, OK185350, OK185351, OK185352, OK185353, OK185354, and SRX12275598.
